# Impact of underlying malignancy on emergency department utilization and outcomes

**DOI:** 10.1002/cam4.4414

**Published:** 2021-11-24

**Authors:** Alexander S. Qian, Edmund M. Qiao, Vinit Nalawade, Rohith S. Voora, Nikhil V. Kotha, Christian Dameff, Christopher J. Coyne, James D. Murphy

**Affiliations:** ^1^ Department of Radiation Medicine and Applied Sciences University of California San Diego La Jolla California USA; ^2^ Department of Emergency Medicine University of California San Diego La Jolla California USA

**Keywords:** emergency department, health services, hospital admission, hospital death

## Abstract

**Purpose:**

Cancer patients frequently utilize the emergency department (ED) for a variety of diagnoses both related to and unrelated to their cancer, yet ED outcomes for cancer patients are not well documented. This study sought to define risks and identify predictors for inpatient admission and hospital mortality among cancer patients presenting to the ED.

**Patients and Methods:**

We utilized the National Emergency Department Sample to identify patients with and without a diagnosis of cancer presenting to the ED between January 2016 and December 2018. We used multivariable mixed‐effects logistic regression models to assess the influence of cancer on outcomes of hospital admission after the ED visit and hospital mortality for the whole patient cohort and individual presenting diagnoses.

**Results:**

There were 340 million weighted ED visits, of which 8.3 million (2.3%) were associated with a cancer diagnosis. Compared to non‐cancer patients, patients with cancer had an increased risk of inpatient admission (64.7% vs. 14.8%; *p* < 0.0001) and hospital mortality (4.6% vs. 0.5%; *p* < 0.0001). For each of the top 15 presenting diagnoses, cancer patients had increased risks of hospitalization (odds ratio [OR] range 2.0–13.2) or death (OR range 2.1–14.4). Although our dataset does not contain reliable estimation of stage, cancer site was the most robust individual predictor associated with the risk of hospitalization or death compared to other clinical or system‐related factors.

**Conclusions:**

Cancer patients in the ED have high risks for hospital admission and death when compared to patients without cancer. Cancer patients represent a distinct population and may benefit from cancer‐specific risk stratification or focused interventions to improve outcomes.

## Graphical abstract

On average, 2.8 million (2.3%) patients visit the emergency department. For each of the top 15 presenting diagnoses cancer patients had increased risks of hospitalization and death compared to non‐cancer patients. Among cancer patients, cancer site was the most robust individual predictor associated with risk of hospitalization or death.

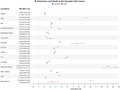

## 1 INTRODUCTION

Cancer represents the second leading cause of death among American adults.^1^ Research demonstrates that a substantial proportion of cancer patients receive care in the emergency department (ED) at some point after their diagnosis, with an estimated 4.2 million visits to the ED per year.^2^ Existing emergency medicine research among cancer patients most often focuses on the management of specific cancer‐related complications or treatment‐related toxicity. However, many ED visits among cancer patients are attributable to common complaints,^2^ which may not directly relate to their cancer or complications from treatment.

Caring for cancer patients in an ED setting ideally utilizes multidisciplinary care, though in particular involves integration of emergency medicine physicians and cancer physicians. An underlying malignancy complicates routine ED management, even with presenting diseases unrelated to a patient's cancer, and a greater comprehension of outcomes and risks among cancer patients presenting to the ED is needed to provide adequate care. Research has worked to characterize utilization of the ED by cancer patients,^2^, ^3^, ^4^ however we lack a large scale study that evaluates the impact of cancer on patient outcomes associated with common presenting conditions within the ED. Understanding the impact of cancer on ED visit outcomes can help raise awareness of specific risks this unique population faces, and could help better focus future interventions aimed at improving outcomes. The purpose of this study was to define the impact of cancer on the likelihood of hospital admission and hospital mortality after an ED visit using a large nationwide emergency database.

## 2 METHODS

### 2.1 Data source

The National Emergency Department Sample (NEDS) database is a nationally representative ED database developed and maintained by Healthcare Cost and Utilization Project as a partnership among federal, state, and industry stakeholders and sponsored by Agency for Healthcare Research and Quality (AHRQ). The database is the largest nationally representative ED database, covering 79.2% of all US ED visits across 37 states and capturing demographic, clinical data, and outcomes of patients seen in community, public, and academic medical centers.

### 2.2 Study population

This study included adults (≥18 years of age) within NEDS who visited the ED between 1 January 2016 and 31 December 2018, the most recent available year. Each ED encounter represented a unique individual visit; repeat visits by the same patient were not able to be identified as no patient identifiers were included in the database. Patients with cancer were identified from International Classification of Diseases, Tenth Revision, Clinical Modification (ICD10‐CM) diagnosis codes (Table S1). The specific cancer site was classified using an established approach based on their first listed cancer diagnoses.^2^ Metastatic disease was determined with secondary malignancy codes, C77–C80. While these codes may have limited sensitivity or specificity,^5^ a sensitivity analysis was conducted where these codes were removed which did not lead to any substantial difference in results (data not shown).

### 2.3 Study variables and outcomes

The following variables were extracted from the NEDS database: age, sex, metropolitan versus rural area, median household income of patient's zip code, primary payer, weekend versus weekday visit, hospital location, teaching status, and trauma center status. Patients presenting to an ED have up to 35 diagnoses listed. In line with existing research we defined the *principal diagnosis* as ICD10‐CM codes listed.^6^, ^7^ A sensitivity analysis was conducted where we defined the principal diagnosis as the first listed non‐cancer ICD10‐CM code,^2^, ^8^ which did not lead to a substantial difference in results (data not shown). Our primary outcomes of interest were inpatient admission to an acute care facility, and overall hospital mortality which included either death in the ED or during inpatient admission. A secondary outcome of interest included preventable readmissions. Preventable hospital admissions were characterized using ICD‐10 codes for Prevention Quality Indicators (PQIs), which are a set of measures, developed by AHRQ, that can be used with hospital inpatient discharge data as a “screening tool” to identify ambulatory conditions for which high‐quality, community‐based outpatient care can potentially prevent hospitalization, complications, or more severe disease (Table S2).

### 2.4 Statistical analysis

We used descriptive statistical analysis to compare patient demographics between cancer versus non‐cancer patients. To determine the impact of cancer diagnosis on our outcomes of inpatient admission and hospital mortality among all patients, we used multivariable logistic mixed‐effect regression models. This analytic approach allowed us to account for clustering of patients within hospital facilities. Variables in each multivariable regression model were identified de novo, and include the list of variables noted above. We determined the impact of cancer on inpatient admission and hospital mortality among all cancer and non‐cancer patients irrespective of primary diagnosis. We hypothesized that the impact cancer had on inpatient admission and hospital mortality would vary by principal diagnosis. Therefore, we identified the 15 top primary diagnosis codes among the cancer patients, created cohorts of all patients with the primary diagnosis, and assessed the impact of cancer separately on outcomes among each cohort with separate multivariable logistic mixed‐effect regression models. To identify cancer patient‐specific risk factors associated with hospital admission and mortality we used a regression analysis on all cancer patients combined into a single cohort. All results were calculated and presented using sample weights to allow us to present nationally representative estimates. Analyses were conducted using R 3.5.1.

## 3 RESULTS

### 3.1 Patient demographics

Between January 2016 and December 2018, we identified 340,554,820 weighted ED visits across 984 hospitals. Among these visits, 8,326,774 (2.39%) were associated with a diagnosis of cancer. Table 1 outlines the demographic characteristics, disposition status, and hospital burden between cancer and non‐cancer visits. Overall, adult patients with cancer were more likely to be elderly, male, and have Medicare insurance compared to non‐cancer patients. Among all ED visits from cancer patients the most frequently reported diagnoses included leukemias and other hematopoietic syndromes (19.6%), metastatic secondary neoplasms (18.7%), and lung cancer (11.7%), followed by gastrointestinal cancers (8.6%), breast cancer (5.4%), and Non‐Hodgkin lymphoma (5.3%) (see Table 1).

**TABLE 1:** Cancer versus non‐cancer patient visit demographics

Demographics	Cancer patients (*N* = 8,326,774) 2.39%	Non‐cancer patients (*N* = 340,554,820) 97.61%
Age		
18–24	73,657 (0.9%)	43,327,008 (12.7%)
25–44	571,940 (6.9%)	117,349,538 (34.5%)
45–64	2,709,925 (32.5%)	99,436,310 (29.2%)
65–74	2,242,344 (26.9%)	36,432,818 (10.7%)
>74	2,728,908 (32.8%)	44,009,146 (12.9%)
Female	4,081,058 (49.0%)	195,065,813 (57.3%)
Weekend	2,086,138 (25.1%)	93,052,742 (27.3%)
Payer		
Medicare	5,055,296 (60.7%)	97,360,829 (28.6%)
Medicaid	1,004,666 (12.1%)	85,575,274 (25.1%)
Private	1,832,123 (22.0%)	96,370,833 (28.3%)
Self‐pay	230,397 (2.8%)	44,753,533 (13.1%)
Income quartile		
1st (lowest)	2,323,571 (27.9%)	117,851,644 (34.6%)
2nd	2,118,949 (25.4%)	91,560,977 (26.9%)
3rd	1,916,888 (23.0%)	69,962,940 (20.5%)
4th (highest)	1,821,225 (21.9%)	54,866,917 (16.1%)
Rural	560,119 (6.7%)	23,822,110 (7.0%)
Teaching hospital	5,453,981 (65.5%)	196,593,259 (57.7%)
Hospital type		
Government (ref)	612,276 (7.4%)	26,152,010 (7.7%)
Private non‐profit	1,857,985 (22.3%)	76,918,895 (22.5%)
Private for profit	491,261 (5.9%)	28,810,317 (8.5%)
Uncategorized	5,365,253 (64.4%)	209,572,583 (61.5%)
Preventable visit	626,572 (7.5%)	26,955,780 (7.9%)
Cancer type		
Leukemia, multiple myeloma, and other hematopoietic syndromes	1,630,091 (19.6%)	
Secondary metastatic neoplasms	1,562,345 (18.7%)	
Lung and other intrathoracic organs	976,173 (11.7%)	
Gastrointestinal	717,322 (8.6%)	
Breast	451,621 (5.4%)	
Non‐Hodgkin lymphoma	439,719 (5.3%)	
Prostate	394,956 (4.7%)	
Female reproductive	303,241 (3.6%)	
Pancreas	267,893 (3.2%)	
Liver	230,485 (2.8%)	
Bladder and other urinary	193,146 (2.3%)	
Head and neck	163,558 (2.0%)	
Brain, nervous system, and eye	147,415 (1.8%)	
Melanoma and other malignant neoplasm of skin	141,370 (1.7%)	
Other^a^	707,439 (8.5%)	

^a^ Other cancers include kidney, bones and connective tissue, active cancer sequelae, Hodgkin lymphoma, other digestive organs, neuroendocrine tumors, thyroid, male reproductive, other endocrine system, and ill‐defined cancers.

**FIGURE 1:**
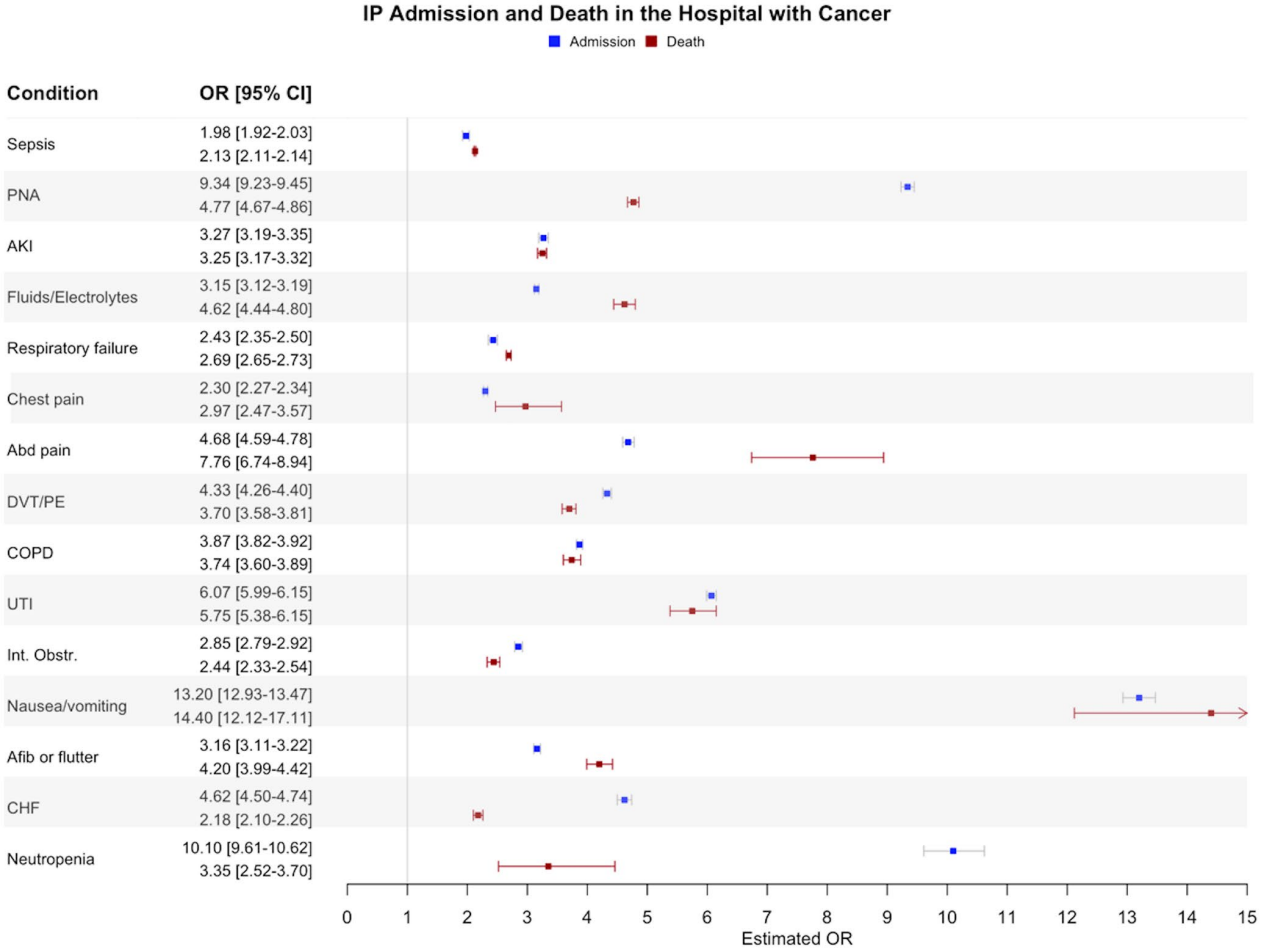
Forest plot displaying results of separate multivariable mixed‐effects logistic regressions evaluating the impact of underlying cancer diagnosis on the risk of inpatient admission and death for the 15 most common ED principal diagnoses among cancer patients. Each condition represents a separate model with dots representing adjusted odds ratios for inpatient admission (blue dots), and death (red dots) for cancer compared to non‐cancer patients. The error bars indicate 95% confidence intervals. Afib, atrial fibrillation; AKI, acute kidney injury; COPD, chronic obstructive pulmonary disease; DVT, deep vein thrombosis; ED, emergency department; PE, pulmonary embolism; UTI, urinary tract infection

Overall, cancer patients had higher rates of hospital admission compared with non‐cancer patients (64.7% vs. 14.8%; *p* < 0.0001) and higher rates of overall hospital mortality (4.6% vs. 0.5%; *p* < 0.0001). On multivariable analysis, having cancer resulted in an adjusted odds ratios (ORs) of 10.1 (95% confidence interval [CI] 10.0–10.1; *p* < 0.0001) for inpatient admission, and 7.6 (95% CI 7.5–7.6; *p* < 0.0001) for death in the hospital compared to not having cancer. Table 2 demonstrates the top 15 most common presenting diagnoses among cancer patients, which together accounted for 36.2% of all presenting diagnoses among cancer patients. The risks of hospital admission and death in the hospital varied by principal diagnosis, though in general the rates were higher among cancer patients compared to non‐cancer patients for each presenting diagnosis. The increased risks of hospital admission and death in the hospital held on multivariable analyses for each individual diagnosis (Figure 1). This increased risk varied by principal diagnosis, though this translated into ORs of ranging from 2.0 to 13.2 for inpatient admission and 2.1–14.4 for hospital death. The highest odds for inpatient admission were for nausea/vomiting (OR = 13.2; 95% CI [13.0–13.5]), and this diagnosis was associated with the highest hospital mortality (OR = 14.4 [12.1–17.1]).

**TABLE 2:** Total number of encounters and outcomes for each principal diagnosis for cancer and non‐cancer patients, ordered by most common principal diagnoses in cancer patients

Principal diagnosis	Total		Fraction admitted		Fraction died in ED or hospital	
	Cancer	Non‐cancer	Cancer	Non‐cancer	Cancer	Non‐cancer
Sepsis	745,024	4,928,874	97.5%	95.0%	16.6%	7.9%
Pneumonia	347,779	5,224,094	85.2%	33.7%	5.5%	0.7%
AKI	221,092	1,518,520	93.8%	83.4%	5.7%	1.6%
Fluid electrolyte imbalance	197,261	2,877,383	55.4%	26.4%	2.1%	0.3%
Respiratory failure	192,400	1,238,807	93.9%	87.5%	21.1%	7.8%
Chest pain	183,145	16,007,013	10.6%	3.6%	0.06%	0.01%
Abdominal pain	167,107	14,452,267	7.5%	1.2%	0.2%	0.01%
DVT/PE	160,000	1,075,464	83.5%	55.6%	4.8%	1.1%
COPD	151,805	3,892,429	76.4%	42.7%	2.2%	0.4%
UTI	143,773	6,463,433	56.8%	12.0%	0.9%	0.07%
Intestinal obstruction	124,410	1,072,941	87.3%	70.9%	2.7%	1.0%
Nausea and vomiting	96,927	4,116,739	20.8%	1.4%	0.3%	0.01%
Atrial fibrillation or flutter	93,874	1,973,206	74.5%	49.8%	2.2%	0.4%
Chronic heart failure	85,446	1,414,996	87.7%	65.1%	4.4%	1.7%
Neutropenia	82,824	19,584	86.1%	46.2%	1.3%	0.4%

Abbreviations: AKI, acute kidney injury; COPD, chronic obstructive pulmonary disease; DVT, deep vein thrombosis; ED, emergency department; PE, pulmonary embolism; UTI, urinary tract infection.

**FIGURE 2:**
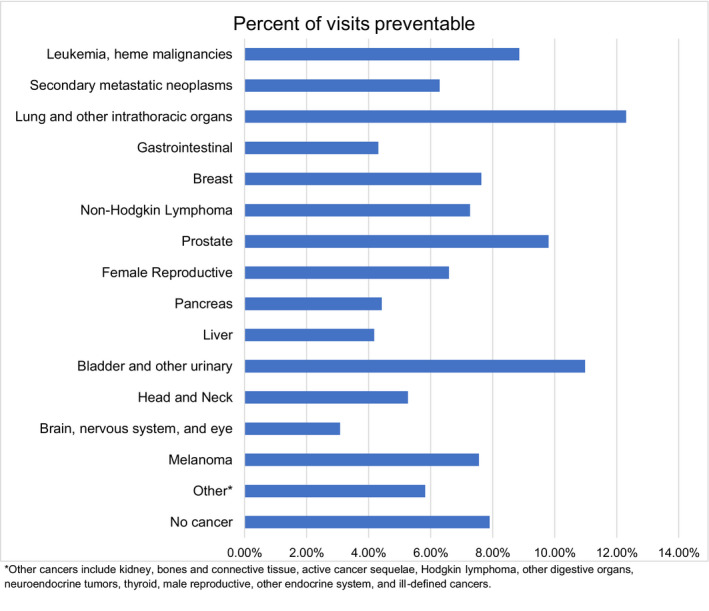
Bar graph depicting % of visits of deemed preventable by AHRQ’s Prevention Quality Indicators. (see Table S2 for PQI definitions). AHRQ, Agency for Healthcare Research and Quality

Analysis pooling of all the cancer patients found that select patient characteristics were associated with an increased risk of hospitalization or increased risk of death (Table 3). Factors associated with both an increased risk of hospitalization and death included older age, male gender, lower income level, discharge quarter, and receipt of care in a teaching hospital. With payer type, patients with private insurance or self‐pay had higher risks of hospitalization, though lower risks of death. The risks of admission and death varied substantially by cancer subsite. Cancers with the highest risk of hospital admission included secondary metastatic, lung, liver, and pancreas. Cancer types with the highest risk of death included secondary metastatic neoplasms, non‐Hodgkin lymphoma, and liver. Breast, prostate, and skin cancers were associated with least risk for hospitalization or death. Lung and bladder cancers had a higher percentage of preventable visits when compared to the non‐cancer population (Figure 2).

**TABLE 3:** Patient characteristics associated with the risk of inpatient admission and death among all cancer patients

Characteristic	Odds ratio of inpatient admission [95% CI]	Odds ratio of death [95% CI]
Age		
18–24	0.65 [0.64–0.67]	0.42 [0.40–0.45]
25–44	0.80 [0.72–0.72]	0.63 [0.62–0.64]
45–64 (ref)	1.00	1.00
65–74	1.15 [1.13–1.16]	1.22 [1.21–1.23]
75+	1.38 [1.36–1.40]	1.47 [1.45–1.48]
Male	1.17 [1.16–1.17]	1.09 [1.07–1.11]
Payer		
Medicare (ref)	1.00	1.00
Medicaid	1.00 [0.99–1.02]	0.86 [0.85–0.87]
Private	1.06 [1.05–1.07]	0.86 [0.86–0.87]
Self‐pay	1.15 [1.12–1.18]	0.71 [0.71–0.72]
Income quartile		
1st	1.02 [1.01–1.03]	1.02 [1.02–1.03]
2nd	1.00 [0.99–1.02]	1.04 [1.03–1.04]
3rd	1.01 [1.00–1.02]	1.02 [1.01–1.03]
4th (ref)	1.00	1.00
Rural hospital	0.99 [0.97–1.01]	0.85 [0.85–0.86]
Hospital type		
Government (ref)	1.00	1.00
Private non‐profit	0.94 [0.90–0.98]	1.02 [1.01–1.02]
Private for profit	0.90 [0.88–0.92]	1.44 [1.11–1.17]
Uncategorized	0.97 [0.96–0.98]	0.93 [0.91–0.96]
Teaching hospital	1.05 [1.03–1.07]	1.05 [1.01–1.09]
Cancer		
Prostate (ref)	1.00	1.00
Leukemia, multiple myeloma, and other hematopoietic syndromes	2.39 [2.33–2.45]	2.54 [2.51–2.56]
Secondary metastatic neoplasms	3.91 [3.83–4.00]	5.71 [5.66–5.76]
Lung and other intrathoracic organs	3.43 [3.35–3.51]	2.25 [2.23–2.27]
Gastrointestinal	2.23 [2.18–2.29]	2.24 [2.22–2.26]
Breast	1.14 [1.10–1.18]	0.97 [0.96–0.98]
Non‐Hodgkin lymphoma	2.25 [2.19–2.31]	2.72 [2.70–2.75]
Female reproductive	1.54 [1.49–1.59]	1.47 [1.45–1.48]
Pancreas	2.89 [2.81–2.97]	2.33 [2.30–2.36]
Liver	3.71 [3.61–3.82]	2.57 [2.54–2.60]
Bladder and other urinary	1.29 [1.24–1.33]	1.78 [1.76–1.80]
Head and neck	1.71 [1.65–1.77]	1.46 [1.44–1.48]
Brain, nervous system, and eye	1.83 [1.77–1.90]	2.37 [2.34–2.40]
Melanoma and other malignant neoplasm of skin	1.12 [1.08–1.17]	1.36 [1.34–1.38]
Other^a^	2.54 [2.48–2.60]	5.19 [5.12–5.26]

Abbreviations: CI, confidence interval; ref, reference.

^a^ Other cancers include kidney, bones and connective tissue, active cancer sequelae, Hodgkin lymphoma, other digestive organs, neuroendocrine tumors, thyroid, male reproductive, other endocrine system, and ill‐defined cancers.

## 4 DISCUSSION

As the population ages and the number of patients with cancer increases,^9^ it is crucial to have a clear understanding of how patients with cancer utilize hospital resources such as the ED. The findings in this current analysis support the existing literature demonstrating increased risks for patients with cancer in the emergency setting. Prior research on individual diseases demonstrates that individuals with a history of cancer have a higher likelihood of inpatient admission as well as a higher risk of mortality.^10^, ^11^, ^12^, ^13^, ^14^ In this nationally representative cohort of ED visits we find that cancer patients face these same risks across a range of presenting conditions. The top presenting illnesses among cancer patients represented both general illnesses as well as complications more directly related to cancer and cancer treatment. Common presenting diagnoses such as pneumonia, congestive heart failure, and chronic obstructive pulmonary disease accounted for a large portion of cancer patient ED visits, though diagnoses commonly associated with cancer including fluid/electrolyte imbalance and neutropenia also contributed considerable visit numbers. Regardless of whether the principal diagnosis was attributable to cancer, the cohort of patients with cancer demonstrated a higher odds of inpatient admission and hospital mortality across all principal diagnoses evaluated.

The underlying reasons for the increased risks of admission and hospital mortality rates among cancer patients are very likely multifactorial, though potential contributing factors deserve discussion. Individual patient attributes that predispose patients to cancer, such as smoking or obesity, tend to increase risks of complications and mortality from a variety of diseases.^15^, ^16^ Additionally, the increased prevalence of comorbidity among cancer patients compared to non‐cancer patients could further contribute to the increased risk of hospital admission and overall mortality.^17^ One must also consider that the nature and severity of presenting diagnoses may fundamentally differ among cancer patients. For example, acute kidney injury secondary to cancer treatment (toxicity from systemic therapy or tumor lysis syndrome) could represent a more precarious manifestation of acute kidney injury which would exclusively impact the cancer population. Additionally, malignancy can potentially obscure presentations of common illnesses which may delay or preclude optimal management.^18^, ^19^ Studies have also described provider differences in treatment choices for cancer and non‐cancer patients as well.^20^, ^21^ For instance, cancer patients may be less likely to receive guideline recommended treatment for myocardial infraction.^22^ In tandem, these patient‐specific and system‐related factors underscore the furtive complexity of caring for cancer patients in the emergency setting. Current methods to identify potentially preventable visits are incomplete, but can still be used as a method to improve hospital outcomes.^23^, ^24^ Utilizing the AHRQ PQI identification codes, lung and bladder cancers had the highest rates of preventable visits likely due to disease‐site related issues such as COPD or UTI, and such patients could potentially benefit from increased outpatient care. Ultimately, deciphering the underlying causes of the increased risks cancer patients face in the emergency setting requires additional research.

A better understanding of the risks cancer patients experience in the emergency setting can identify individuals most at risk (risk stratification), and potentially help guide future interventions. Our study highlights important associations between patient characteristics and the risks of inpatient admission and hospital mortality. Our study parallels existing research on predictors of ED admission for general patients,^25^, ^26^ though we demonstrate notable differences. Specifically, we demonstrate that cancer type represents the most robust predictor (with the largest effect sizes) for both inpatient admission and hospital death among cancer patients. Our analysis demonstrates that select cancer types have up to a 5× increased odds of admission or death compared to other cancer types. These different risks by cancer type likely represent inherent differences between cancers with respect to severity, treatment, or frequency of comorbid illness. For example, our analysis found that liver cancer was associated with a high risk of hospital admission and death. The majority of patients with liver cancer have concurrent underlying liver disease including hepatitis, non‐alcoholic fatty liver disease, or cirrhosis which very likely impacts a patient's risk of hospital admission or death. Research demonstrates that cancer‐specific pathways in general within an ED triage system can improve outcomes in terms of inpatient admission, decreased ED visits, and decreased overall mortality.^27^, ^28^, ^29^ Given the variable outcomes among different cancer subtypes our study underscores importance of considering cancer type in the risk stratification process of this uniquely vulnerable patient population.

This current study has a limitations worth mentioning. First, this study used ICD10 codes to identify cancer patients, cancer types, primary diagnoses, and presence of metastatic disease. Misclassification of these variables represents a potential limitation of this study which could introduce bias into our analysis. While we suspect the misclassification would occur at random, which would attenuate our findings (i.e., produce results that underestimate the true effects), further study with independent validation is necessary. Second, the large administrative dataset used in this current study lacks important data including information about tumor stage and extent of disease, treatment characteristics, and date of cancer diagnosis. Thus, certain patients with advanced disease but no secondary coding of metastasis would be included in their respective cancer cohort instead of metastatic disease. Additionally, clinical characteristics such as performance status, medication use, and comorbidity were not included in our analysis. Finally, we lack detailed information about the ED visit including specific symptoms, lab, test, imaging, or other diagnostic workup information. We expect that all of these factors would influence the risk of hospitalization and death, and if available would further refine our risk prediction. Additional studies with more granular data are needed to further characterize the impact of these relevant variables on our endpoints.

Given the high prevalence of cancer‐related emergency visits there exists an imperative to effectively triage and management cancer patients who visit the ED. This study attempts to comprehensively characterize the risks among cancer patients presenting to the ED. Overall, we found that a cancer history in general significantly increased the risks of hospital admission and hospital death, with risk varying substantially by cancer subsite. These findings are relevant for both emergency physicians and oncology providers seeking to help risk‐stratify cancer patients, and could also prove valuable in future interventional research seeking to improve outcomes among cancer patients presenting to the ED.

## CONFLICT OF INTEREST

JM receives compensation for consulting from Boston Consulting Group.

## ETHICAL APPROVAL STATEMENT

The authors are accountable for all aspects of the work in ensuring that questions related to the accuracy or integrity of any part of the work are appropriately investigated and resolved. All procedures performed in studies involving human participants were in accordance with the ethical standards of the institutional and/or national research committee(s) and with the Helsinki Declaration (as revised in 2013). Written informed consent was obtained from the patient.

## Supporting information

TABLE S1

TABLE S2

## Data Availability

Cancer Medicine expects that data supporting the results in the paper will be archived in an appropriate public repository. The authors are required to provide a data availability statement to describe the availability or the absence of shared data. When data have been shared, authors are required to include in their data availability statement a link to the repository they have used, and to cite the data they have shared. Whenever possible the scripts and other artifacts used to generate the analyses presented in the paper should also be publicly archived. If sharing data compromises ethical standards or legal requirements then authors are not expected to share it.
