# Supplementary material for: Impact of underlying malignancy on emergency department utilization and outcomes
Source: Cancer Med. 2021 Nov 24;10(24):9129–38. doi: 10.1002/cam4.4414 (PMC8683529; doi:10.1002/cam4.4414)
Supplement: Supplementary file 1 — TABLE S1 [file CAM4-10-9129-s002.docx]

**Supplemental Table 1:** ICD10 codes for cancers

| Head and Neck | C00 to C14, C32 |
| --- | --- |
| Gastrointestinal | C15, C16, C17, C18, C19, C20, C21 |
| Liver | C22 |
| Pancreas | C25 |
| Other digestive organ | C23, C24, C26 |
| Lung and other respiratory/intrathoracic organs | C33, C34, C30, C31, C37, C38, C39, C45 |
| Other respiratory and intrathoracic organs | C30, C31, C37, C38, C39, C45 |
| Bones and connective tissue | C40, C41, C48, C49 |
| Melanoma and other malignant neoplasm of skin | C43, C44, C46 |
| Breast | C50 |
| Female reproductive | C53, C54, C55, C56, C51, C52, C57, C58 |
| Male reproductive | C60, C62, C63 |
| Prostate | C61 |
| Kidney | C64, C65, C66 |
| Bladder and other urinary | C67, C68 |
| Brain and nervous system and eye | C70, C71, C72, C47, C69 |
| Thyroid | C73 |
| Other endocrine system | C74, C75 |
| Non-Hodgkin Lymphoma | C82, C83, C841-C849, C85, C86 |
| Hodgkin Lymphoma | C81 |
| Multiple Myeloma | C88 C90 |
| Leukemias | C91, C92, C93, C94, C95 |
| Ill Defined states | C76 |
| Myelodysplastic syndrome and other hematopoietic | C96, D46, D47, C840 |
| Neuroendocrine tumors | C7A |
| Secondary Neoplasms | C77, C78, C79, C80 |
| Active Cancer sequelae | D630, D6481, E340, E883, G131, G731, J910, K1233, K627, M360, M361, M845, M906, O9A1, R180, R530, T66, Y632, Y842 |
