# Supplementary material for: Impact of underlying malignancy on emergency department utilization and outcomes
Source: Cancer Med. 2021 Nov 24;10(24):9129–38. doi: 10.1002/cam4.4414 (PMC8683529; doi:10.1002/cam4.4414)
Supplement: Supplementary file 2 — TABLE S2 [file CAM4-10-9129-s001.docx]

**Supplemental Table 2:** AHRQ Prevention Quality Indicators with their definition and relative inclusion and exclusion ICD10-CM and ICD10-PCS codes.

| **PQI Number** | **Preventive Quality Indicator** | **Definition** | **ICD-10 Codes** | **ICD-10 Procedure codes** |
| --- | --- | --- | --- | --- |
| 1 | Diabetes short term complications | Discharges with a principal ICD-10- CM diagnosis for diabetes short- term complications (ketoacidosis,  hyperosmolarity, or coma) | E1010, E1011, E10641, E1065, E1300, E1301, E1310, E1311, E13641 |  |
| 3 | Diabetes long term complications | Discharges with a principal ICD-10- CM diagnosis code for diabetes with long-term complications (renal, eye, neurological, circulatory, or complications not otherwise specified) | E10349, E10351, E10359, E1036, E1039, E1040, E1041, E1042, E1043, E1044, E1049, E1051, E1052, E1059, E10610, E10618, E10620, E10621, E10622, E10628, E10630, E10638, E1069, E108, E1121, E1122, E1129, E11311, E11319, E11321, E11329, E11331, E11339, E11341, E11349, E11351, E11359, E1136, E1139, E1140, E1141, E1142, E1143, E1144, E1149, E1151, E1152, E1159, E11610, E11618, E11620, E11621, E11622, E11628, E11630, E11638, E1169, E118, E1321, E1322, E1329, E13311, E13319, E13321, E13329, E13331, E13339, E13341, E13349, E13351, E13359, E1336, E1339, E1340, E1341, E1342, E1343, E1344, E1349, E1351, E1352, E1359, E13610, E13618, E13620, E13621, E13622, E13628, E13630, E13638, E13649, E1365, E1369, E138 |  |
| 5 | Chronic obstructive pulmonary disease or Asthma in Older Adults | ICD 10 for COPD or | J410, J411, J418, J42, J430, J431, J432, J438, J439, J440, J441, J449, J470, J471, J479 |  |
|  |  | ICD 10 for adult asthma | J452, J4521, J4522, J4530, J4531, J4532, J4540, J4541, J4542, J4550, J4551, J4552, J45901, J45902, J45909, J45990, J45991, J45998 |  |
|  |  | Excluding cases: Any listed ICD for cystic fibrosis | E840, E8411, E8419, E848, E849, E8483, E84841, E849, J8483, J84841, J84842, J84843, J84848, P270, P271, P278, P279, Q254, Q311, Q312, Q313, Q315, Q318, Q319, Q320, Q321, Q322, Q323, Q324, Q33, Q34, Q39, Q693 |  |
| 7 | Hypertension | ICD10 for hypertension | I10, I119, I129, I1310 |  |
|  |  | Excluded cases: Any with ICD 10 diagnosis of stage I-IV kidney disease IF accompanied by a ICD10 procedural code for dialysis access | I129, I1310 | 031509D, 031509D, 03170ZV, 03170ZV, 031509F, 031509F, 03170ZW, 03170ZW, 031509V, 031509V, 031809D, 031809D, 031509W, 031509W, 031809F, 031809F, 03150AD, 03150AD, 031809V, 031809V, 03150AF, 03150AF, 031809W, 031809W, 03150AV, 03150AV, 03180AD, 03180AD, 03150AW, 03150AW, 03180AF, 03180AF, 03150JD, 03150JD, 03180AV, 03180AV, 03150JF, 03150JF, 03180AW, 03180AW, 03150JV, 03150JV, 03180JD, 03180JD, 03150JW, 03150JW, 03180JF, 03180JF, 03150KD, 03150KD, 03180JV, 03180JV, 03150KF, 03150KF, 03180JW, 03180JW, 03150KV, 03150KV, 03180KD, 03180KD, 03150KW, 03150KW, 03180KF, 03180KF, 03150ZD, 03150ZD, 03180KV, 03180KV, 03150ZF, 03150ZF, 03180KW, 03180KW, 03150ZV, 03150ZV, 03180ZD, 03180ZD, 03150ZW, 03150ZW, 03180ZF, 03180ZF, 031609D, 031609D, 03180ZV, 03180ZV, 031609F, 031609F, 03180ZW, 03180ZW, 031609V, 031609V, 031909F, 031909F, 031609W, 031609W, 03190AF, 03190AF, 03160AD, 03160AD, 03190JF, 03190JF, 03160AF, 03160AF, 03190KF, 03190KF, 03160AV, 03160AV, 03190ZF, 03190ZF, 03160AW, 03160AW, 03193ZF, 03193ZF, 03160JD, 03160JD, 031A09F, 031A09F, 03160JF, 03160JF, 031A0AF, 031A0AF, 03160JV, 03160JV, 031A0JF, 031A0JF, 03160JW, 03160JW, 031A0KF, 031A0KF, 03160KD, 03160KD, 031A0ZF, 031A0ZF, 03160KF, 03160KF, 031A3ZF, 031A3ZF, 03160KV, 03160KV, 031B09F, 031B09F, 03160KW, 03160KW, 031B0AF, 031B0AF, 03160ZD, 03160ZD, 031B0JF, 031B0JF, 03160ZF, 03160ZF, 031B0KF, 031B0KF, 03160ZV, 03160ZV, 031B0ZF, 031B0ZF, 03160ZW, 03160ZW, 031B3ZF, 031B3ZF, 031709D, 031709D, 031C09F, 031C09F, 031709F, 031709F, 031C0AF, 031C0AF, 031709V, 031709V, 031C0JF, 031C0JF, 031709W, 031709W, 031C0KF, 031C0KF, 03170AD, 03170AD, 031C0ZF, 031C0ZF, 03170AF, 03170AF, 031C3ZF, 031C3ZF, 03170AV, 03170AV, 041K09S, 041K09S, 03170AW, 03170AW, 041K0AS, 041K0AS, 03170JD, 03170JD, 041K0JS, 041K0JS, 03170JF, 03170JF, 041K0KS, 041K0KS, 03170JV, 03170JV, 041K0ZS, 041K0ZS, 03170JW, 03170JW, 041K3JS, 041K3JS, 03170KD, 03170KD, 041L09S, 041L09S, 03170KF, 03170KF, 041L0AS, 041L0AS, 03170KV, 03170KV, 041L0JS, 041L0JS, 03170KW, 03170KW, 041L0KS, 041L0KS, 03170ZD, 03170ZD, 041L0ZS, 041L0ZS, 03170ZF, 03170ZF, 041L3JS, 041L3JS‚Äô |
| 8 | Congestive heart failure | ICD10 diagnosis for CHF: | I0981, I501, I5020, I5021, I5022, I5023, I5030, I5031, I5032, I50.33, I5040, I5041, I50.42, I5043, I509 |  |
| Special exclusions PQI 7 and 8 |  | Excluded cases for PQI 7 and 8: Any with ICD 10 procedural code of cardiac access |  | 02C03ZZ, 0210088, 02C04Z6, 0210089, 02C04ZZ, 021008C, 02C10Z6, 021008F, 02C10ZZ, 021008W, 02C13Z6, 0210093, 02C13ZZ, 0210098, 02C14Z6, 0210099, 02C14ZZ, 021009C, 02C20Z6, 021009F, 02C20ZZ, 021009W, 02C23Z6, 02100A3, 02C23ZZ, 02100A8, 02C24Z6, 02100A9, 02C24ZZ, 02100AC, 02C30Z6, 02100AF, 02C30ZZ, 02100AW, 02C33Z6, 02100J3, 02C33ZZ, 02100J8, 02C34Z6, 02100J9, 02C34ZZ, 02100JC, 02C40ZZ, 02100JF, 02C43ZZ, 02100JW, 02C44ZZ, 02100K3, 02C50ZZ, 02100K8, 02C53ZZ, 02100K9, 02C54ZZ, 02100KC, 02C60ZZ, 02100KF, 02C64ZZ, 02100KW, 02C70ZZ, 02100Z3, 02C74ZZ, 02100Z8, 02C80ZZ, 02100Z9, 02C84ZZ, 02100ZC, 02C90ZZ, 02100ZF, 02C94ZZ, 0210344, 02CD0ZZ, 02103D4, 02CD3ZZ, 0210444, 02CD4ZZ, 0210483, 02CF0ZZ, 0210488, 02CF3ZZ, 0210489, 02CF4ZZ, 021048C, 02CG0ZZ, 021048F, 02CG3ZZ, 021048W, 02CG4ZZ, 0210493, 02CH0ZZ, 0210498, 02CH3ZZ, 0210499, 02CH4ZZ, 021049C, 02CJ0ZZ, 021049F, 02CJ3ZZ, 021049W, 02CJ4ZZ, 02104A3, 02CK0ZZ, 02104A8, 02CK4ZZ, 02104A9, 02CL0ZZ, 02104AC, 02CL4ZZ, 02104AF, 02CM0ZZ, 02104AW, 02CM3ZZ, 02104D4, 02CM4ZZ, 02104J3, 02CN0ZZ, 02104J8, 02H00DZ, 02104J9, 02H00YZ, 02104JC, 02H03DZ, 02104JF, 02H03YZ, 02104JW, 02H04DZ, 02104K3, 02H04YZ, 02104K8, 02H10DZ, 02104K9, 02H10YZ, 02104KC, 02H13DZ, 02104KF, 02H13YZ, 02104KW, 02H14DZ, 02104Z3, 02H14YZ, 02104Z8, 02H20DZ, 02104Z9, 02H20YZ, 02104ZC, 02H23DZ, 02104ZF, 02H23YZ, 0211083, 02H24DZ, 0211088, 02H24YZ, 0211089, 02H30DZ, 021108C, 02H30YZ, 021108F, 02H33DZ, 021108W, 02H33YZ, 0211093, 02H34DZ, 0211098, 02H34YZ, 0211099, 02H400Z, 021109C, 02H402Z, 021109F, 02H403Z, 021109W, 02H40DZ, 02110A3, 02H40JZ, 02110A8, 02H40KZ, 02110A9, 02H40MZ, 02110AC, 02H40NZ, 02110AF, 02H430Z, 02110AW, 02H432Z, 02110J3, 02H433Z, 02110J8, 02H43DZ, 02110J9, 02H43JZ, 02110JC, 02H43KZ, 02110JF, 02H43MZ, 02110JW, 02H43NZ, 02110K3, 02H440Z, 02110K8, 02H442Z, 02110K9, 02H443Z, 02110KC, 02H44DZ, 02110KF, 02H44JZ, 02110KW, 02H44KZ, 02110Z3, 02H44MZ, 02110Z8, 02H44NZ, 02110Z9, 02H600Z, 02110ZC, 02H60JZ, 02110ZF, 02H60KZ, 0211344, 02H60MZ, 02113D4, 02H60NZ, 0211444, 02H630Z, 0211483, 02H63JZ, 0211488, 02H63KZ, 0211489, 02H63MZ, 021148C, 02H640Z, 021148F, 02H64JZ, 021148W, 02H64KZ, 0211493, 02H64MZ, 0211498, 02H64NZ, 0211499, 02H700Z, 021149C, 02H70JZ, 021149F, 02H70KZ, 021149W, 02H70MZ, 02114A3, 02H70NZ, 02114A8, 02H730Z, 02114A9, 02H73JZ, 02114AC, 02H73KZ, 02114AF, 02H73MZ, 02114AW, 02H740Z, 02114D4, 02H74JZ, 02114J3, 02H74KZ, 02114J8, 02H74MZ, 02114J9, 02H74NZ, 02114JC, 02HA0QZ, 02114JF, 02HA0RJ, 02114JW, 02HA0RS, 02114K3, 02HA0RZ, 02114K8, 02HA3QZ, 02114K9, 02HA3RJ, 02114KC, 02HA3RS, 02114KF, 02HA3RZ, 02114KW, 02HA4QZ, 02114Z3, 02HA4RJ, 02114Z8, 02HA4RS, 02114Z9, 02HA4RZ, 02114ZC, 02HK00Z, 02114ZF, 02HK02Z, 0212083, 02HK0JZ, 0212088, 02HK0KZ, 0212089, 02HK0MZ, 021208C, 02HK0NZ, 021208F, 02HK30Z, 021208W, 02HK32Z, 0212093, 02HK3JZ, 0212098, 02HK3KZ, 0212099, 02HK3MZ, 021209C, 02HK40Z, 021209F, 02HK42Z, 021209W, 02HK4JZ, 02120A3, 02HK4KZ, 02120A8, 02HK4MZ, 02120A9, 02HK4NZ, 02120AC, 02HL00Z, 02120AF, 02HL0JZ, 02120AW, 02HL0KZ, 02120J3, 02HL0MZ, 02120J8, 02HL0NZ, 02120J9, 02HL30Z, 02120JC, 02HL3JZ, 02120JF, 02HL3KZ, 02120JW, 02HL3MZ, 02120K3, 02HL40Z, 02120K8, 02HL4JZ, 02120K9, 02HL4KZ, 02120KC, 02HL4MZ, 02120KF, 02HL4NZ, 02120KW, 02HN0JZ, 02120Z3, 02HN0KZ, 02120Z8, 02HN0MZ, 02120Z9, 02HN3JZ, 02120ZC, 02HN3KZ, 02120ZF, 02HN3MZ, 0212344, 02HN4JZ, 02123D4, 02HN4KZ, 0212444, 02HN4MZ, 0212483, 02HS00Z, 0212488, 02HS30Z, 0212489, 02HS40Z, 021248C, 02HT00Z, 021248F, 02HT30Z, 021248W, 02HT40Z, 0212493, 02HV00Z, 0212498, 02HV30Z, 0212499, 02HV40Z, 021249C, 02HX02Z, 021249F, 02HX0DZ, 021249W, 02HX42Z, 02124A3, 02HX4DZ, 02124A8, 02L70CK, 02124A9, 02L70DK, 02124AC, 02L70ZK, 02124AF, 02L73CK, 02124AW, 02L73DK, 02124D4, 02L73ZK, 02124J3, 02L74CK, 02124J8, 02L74DK, 02124J9, 02L74ZK, 02124JC, 02LQ0CZ, 02124JF, 02LQ0DZ, 02124JW, 02LQ0ZZ, 02124K3, 02LQ3CZ, 02124K8, 02LQ3DZ, 02124K9, 02LQ3ZZ, 02124KC, 02LQ4CZ, 02124KF, 02LQ4DZ, 02124KW, 02LQ4ZZ, 02124Z3, 02LR0CZ, 02124Z8, 02LR0DZ, 02124Z9, 02LR0ZT, 02124ZC, 02LR0ZZ, 02124ZF, 02LR3CZ, 0213083, 02LR3DZ, 0213088, 02LR3ZZ, 0213089, 02LR4CZ, 021308C, 02LR4DZ, 021308F, 02LR4ZZ, 021308W, 02LS0ZZ, 0213093, 02LT0ZZ, 0213098, 02N00ZZ, 0213099, 02N03ZZ, 021309C, 02N04ZZ, 021309F, 02N10ZZ, 021309W, 02N13ZZ, 02130A3, 02N14ZZ, 02130A8, 02N20ZZ, 02130A9, 02N23ZZ, 02130AC, 02N24ZZ, 02130AF, 02N30ZZ, 02130AW, 02N33ZZ, 02130J3, 02N34ZZ, 02130J8, 02N50ZZ, 02130J9, 02N53ZZ, 02130JC, 02N54ZZ, 02130JF, 02N90ZZ, 02130JW, 02N93ZZ, 02130K3, 02N94ZZ, 02130K8, 02ND0ZZ, 02130K9, 02ND3ZZ, 02130KC, 02ND4ZZ, 02130KF, 02NF0ZZ, 02130KW, 02NF3ZZ, 02130Z3, 02NF4ZZ, 02130Z8, 02NG0ZZ, 02130Z9, 02NG3ZZ, 02130ZC, 02NG4ZZ, 02130ZF, 02NH0ZZ, 0213344, 02NH3ZZ, 02133D4, 02NH4ZZ, 0213444, 02NJ0ZZ, 0213483, 02NJ3ZZ, 0213488, 02NJ4ZZ, 0213489, 02NK0ZZ, 021348C, 02NK3ZZ, 021348F, 02NK4ZZ, 021348W, 02NL0ZZ, 0213493, 02NL3ZZ, 0213498, 02NL4ZZ, 0213499, 02NM0ZZ, 021349C, 02NM3ZZ, 021349F, 02NM4ZZ, 021349W, 02PA0MZ, 02134A3, 02PA0NZ, 02134A8, 02PA0QZ, 02134A9, 02PA0RS, 02134AC, 02PA0RZ, 02134AF, 02PA3MZ, 02134AW, 02PA3QZ, 02134D4, 02PA3RS, 02134J3, 02PA3RZ, 02134J8, 02PA4MZ, 02134J9, 02PA4NZ, 02134JC, 02PA4QZ, 02134JF, 02PA4RS, 02134JW, 02PA4RZ, 02134K3, 02PAXMZ, 02134K8, 02Q00ZZ, 02134K9, 02Q03ZZ, 02134KC, 02Q04ZZ, 02134KF, 02Q10ZZ, 02134KW, 02Q13ZZ, 02134Z3, 02Q14ZZ, 02134Z8, 02Q20ZZ, 02134Z9, 02Q23ZZ, 02134ZC, 02Q24ZZ, 02134ZF, 02Q30ZZ, 021608P, 02Q33ZZ, 021608Q, 02Q34ZZ, 021608R, 02Q40ZZ, 021609P, 02Q43ZZ, 021609Q, 02Q44ZZ, 021609R, 02Q50ZZ, 02160AP, 02Q53ZZ, 02160AQ, 02Q54ZZ, 02160AR, 02Q70ZZ, 02160JP, 02Q73ZZ, 02160JQ, 02Q74ZZ, 02160JR, 02Q90ZZ, 02160KP, 02Q93ZZ, 02160KQ, 02Q94ZZ, 02160KR, 02QA0ZZ, 02160Z7, 02QA3ZZ, 02160ZP, 02QA4ZZ, 02160ZQ, 02QB0ZZ, 02160ZR, 02QB3ZZ, 02163Z7, 02QB4ZZ, 021648P, 02QC0ZZ, 021648Q, 02QC3ZZ, 021648R, 02QC4ZZ, 021649P, 02QD0ZZ, 021649Q, 02QD3ZZ, 021649R, 02QD4ZZ, 02164AP, 02QF0ZJ, 02164AQ, 02QF0ZZ, 02164AR, 02QF3ZJ, 02164JP, 02QF3ZZ, 02164JQ, 02QF4ZJ, 02164JR, 02QF4ZZ, 02164KP, 02QG0ZE, 02164KQ, 02QG0ZZ, 02164KR, 02QG3ZE, 02164Z7, 02QG3ZZ, 02164ZP, 02QG4ZE, 02164ZQ, 02QG4ZZ, 02164ZR, 02QH0ZZ, 021708P, 02QH3ZZ, 021708Q, 02QH4ZZ, 021708R, 02QJ0ZG, 021709P, 02QJ0ZZ, 021709Q, 02QJ3ZG, 021709R, 02QJ3ZZ, 021709S, 02QJ4ZG, 021709T, 02QJ4ZZ, 021709U, 02QM0ZZ, 02170AP, 02QM3ZZ, 02170AQ, 02QM4ZZ, 02170AR, 02R907Z, 02170AS, 02R908Z, 02170AT, 02R90JZ, 02170AU, 02R90KZ, 02170JP, 02R947Z, 02170JQ, 02R948Z, 02170JR, 02R94JZ, 02170JS, 02R94KZ, 02170JT, 02RD07Z, 02170JU, 02RD08Z, 02170KP, 02RD0JZ, 02170KQ, 02RD0KZ, 02170KR, 02RD47Z, 02170KS, 02RD48Z, 02170KT, 02RD4JZ, 02170KU, 02RD4KZ, 02170ZP, 02RF07Z, 02170ZQ, 02RF08Z, 02170ZR, 02RF0JZ, 02170ZS, 02RF0KZ, 02170ZT, 02RF37H, 02170ZU, 02RF37Z, 021748P, 02RF38H, 021748Q, 02RF38Z, 021748R, 02RF3JH, 021749P, 02RF3JZ, 021749Q, 02RF3KH |
| 11 | Bacterial Pneumonia | ICD 10 diagnosis for PNA: | J13, J14, J15211, J15212, J153, J154, J157, J159, J160, J168, J180, J181, J188, J189 |  |
|  |  | Exclusion ICD10 for sickle cell | D57 |  |
| 12 | Urinary tract infection | ICD10 code for UTI: | N10, N119, N12, N136, N151, N16, N2884, N2885, N2886, N3000, N3001, N3090, N390 |  |
|  |  | Exclusion ICD10: | N110, N111, N118, N1370, N1371, N13721, N13722, N13729, N13731, N13732, N13739, N139, Q600, Q601, Q602, Q603, Q604, Q605, Q606, Q61, Q62, Q63, Q64 |  |
| Special Exclusions PQI11 and 12 |  | Excluded cases for PQI 11 and 12: Any with ICD 10 diagnosis code or ICD 10 procedural code that indicate immunocomprimised state | B20, E41, B59, E42, C802, E43, C888, I120, C9440, I1311, C9441, I132, C9442, K912, C946, N185, D4622, N186, D4701, T8600, D4702, T8601, D4709, T8602, D471, T8603, D479, T8609, D47Z1, T8610, D47Z2, T8611, D47Z9, T8612, D6109, T8613, D61810, T8619, v2020, D61811, T8620, D61818, T8621, D700, T8622, D701, T8623, D702, T86290, D704, T86298, D708, T8630, D709, T8631, D71, T8632, D720, T8633, D72810, T8639, D72818, T8640, D72819, T8641, D7381, T8642, D7581, T8643, D761, T8649, D762, T865, D763, T86810, D800, T86811, D801, T86812, D802, T86818, D803, T86819, D804, T86830, D805, T86831, D806, T86832, D807, T86838, D808, T86839, v2020, D809, T86850, D810, T86851, D811, T86852, D812, T86858, D8130, T86859, D8131, T86890, D8132, T86891, D8139, T86892, D814, T86898, D816, T86899, D817, T8690, D8189, T8691, D819, T8692, D820, T8693, D821, T8699, D822, Z4821, D823, Z4822, D824, Z4823, D828, Z4824, D829, Z48280, v2020, D830, Z48288, D831, Z48290, D832, Z48298, D838, Z4901, D839, Z4902, D840, Z4931, D841, Z4932, D848, Z940, D849, Z941, D893, Z942, D89810, Z943, D89811, Z944, D89812, Z9481, D89813, Z9482, D8982, Z9483, D8989, Z9484, D899, Z9489, E40, Z992 | 30233Y4, 02YA0Z2, 30240AZ, 0BYC0Z0, 30240G0, 0BYC0Z2, 30240G1, 0BYD0Z0, 30240G2, 0BYD0Z2, 30240G3, 0BYF0Z0, 30240G4, 0BYF0Z2, 30240U2, 0BYG0Z0, 30240U3, 0BYG0Z2, 30240U4, 0BYH0Z0, 30240X0, 0BYH0Z2, 30240X1, 0BYJ0Z0, 30240X2, 0BYJ0Z2, 30240X3, 0BYK0Z0, 30240X4, 0BYK0Z2, 30240Y0, 0BYL0Z0, 30240Y1, 0BYL0Z2, 30240Y2, 0BYM0Z0, 30240Y3, 0BYM0Z2, 30240Y4, 0DY50Z0, 30243AZ, 0DY50Z2, 30243G0, 0DY60Z0, 30243G1, 0DY60Z2, 30243G2, 0DY80Z0, 30243G3, 0DY80Z2, 30243G4, 0DYE0Z0, 30243U2, 0DYE0Z2, 30243U3, 0FY00Z0, 30243U4, 0FY00Z2, 30243X0, 0FYG0Z0, 30243X1, 0FYG0Z2, 30243X2, 0TY00Z0, 30243X3, 0TY00Z2, 30243X4, 0TY10Z0, 30243Y0, 0TY10Z2, 30243Y1, 0WY20Z0, 30243Y2, 0XYJ0Z0, 30243Y3, 0XYK0Z0, 30243Y4, 30230AZ, 3E03005, 30230G0, 3E0300M, 30230G1, 3E0300P, 30230G2, 3E030U1, 30230G3, 3E030WL, 30230G4, 3E03305, 30230U2, 3E0330M, 30230U3, 3E0330P, 30230U4, 3E033U1, 30230X0, 3E033WL, 30230X1, 3E04005, 30230X2, 3E0400M, 30230X3, 3E0400P, 30230X4, 3E040WL, 30230Y0, 3E04305, 30230Y1, 3E0430M, 30230Y2, 3E0430P, 30230Y3, 3E043WL, 30230Y4, 3E0A305, 30233AZ, 3E0A30M, 30233G0, 3E0D705, 30233G1, 3E0G705, 30233G2, 3E0J3U1, 30233G3, 3E0J7U1, 30233G4, 3E0J8U1, 30233U2, XW03351, 30233U3, XW033B3, 30233U4, XW033Q5, 30233X0, XW033S5, 30233X1, XW04351, 30233X2, XW043B3, 30233X3, XW043Q5, 30233X4, XW043S5, 30233Y0, XW0DXJ5, 30233Y1, XW0DXL5, 30233Y2, XW0DXR5, 30233Y3, XW0DXT5, XW0DXV5 |
| 14 | Uncontrolled diabetes | ICD10 for uncontrolled diabetes | E1065, E1165, E10649, E11649 |  |
| 15 | Asthma in younger adults | ICD10 for asthma in younger adults: | J4520, J4521, J4522, J4530, J4531, J4532, J4540, J4541, J4542, J4550, J4552, J45901, J45902, J45909, J45990, J45991, J45998 |  |
|  |  | Excluding cases: Any listed ICD for cystic fibrosis | E840, E8411, E8419, E848, E849, E8483, E84841, E849, J8483, J84841, J84842, J84843, J84848, P270, P271, P278, P279, Q254, Q311, Q312, Q313, Q315, Q318, Q319, Q320, Q321, Q322, Q323, Q324, Q33, Q34, Q39, Q693 |  |
| 16 | Lower-extremity amputation among diabetics | ICD10 procedural code for amputation WITH ICD10 code for diabetes (must have a diagnosis from each) | E1010, E1011, E1021, E1022, E1029, E10311, E10319, E10321, E10329, E10331, E10339, E10341, E10349, E10351, E10359, E1036, E1039, E1040, E1041, E1042, E1043, E1044, E1049, E1051, E1052, E1059, E10610, E10618, E10620, E10621, E10622, E10628, E10630, E10638, E10641, E10649, E1065, E1069, E108, E109, E1100, E1101, E1121, E1122, E1129, E11311, E11319, E11321, E11329, E11331, E11339, E11341, E11349, E11351, E11359, E1136, E1139, E1140, E1141, E1142, E1143, E1144, E1149, E1151, E1152, E1159, E11610, E11618, E11620, E11621, E11622, E11628, E11630, E11638, E11641, E11649, E1165, E1169, E118, E119, E1300, E1301, E1310, E1311, E1321, E1322, E1329, E13311, E13319, E13321, E13329, E13331, E13339, E13341, E13349, E13351, E13359, E1336, E1339, E1340, E1341, E1342, E1343, E1344, E1349, E1351, E1352, E1359, E13610, E13618, E13620, E13621, E13622, E13628, E13630, E13638, E13641, E13649, E1365, E1369, E138, E139 | 0Y620ZZ, 0Y630ZZ, 0Y640ZZ, 0Y670ZZ, 0Y680ZZ, 0Y6C0Z1, 0Y6C0Z2, 0Y6C0Z3, 0Y6D0Z1, 0Y6D0Z2, 0Y6D0Z3, 0Y6F0ZZ, 0Y6G0ZZ, 0Y6H0Z1, 0Y6H0Z2, 0Y6H0Z3, 0Y6J0Z1, 0Y6J0Z2, 0Y6J0Z3, 0Y6M0Z0, 0Y6M0Z4, 0Y6M0Z5, 0Y6M0Z6, 0Y6M0Z7, 0Y6M0Z8, 0Y6M0Z9, 0Y6M0ZB, 0Y6M0ZC, 0Y6M0ZD, 0Y6M0ZF, 0Y6N0Z0, 0Y6N0Z4, 0Y6N0Z5, 0Y6N0Z6, 0Y6N0Z7, 0Y6N0Z8, 0Y6N0Z9, 0Y6N0ZB, 0Y6N0ZC, 0Y6N0ZD, 0Y6N0ZF, 0Y6P0Z0, 0Y6P0Z1, 0Y6P0Z2, 0Y6P0Z3, 0Y6Q0Z0, 0Y6Q0Z1, 0Y6Q0Z2, 0Y6Q0Z3, 0Y6R0Z0, 0Y6R0Z1, 0Y6R0Z2, 0Y6R0Z3, 0Y6S0Z0, 0Y6S0Z1, 0Y6S0Z2, 0Y6S0Z3, 0Y6T0Z0, 0Y6T0Z1, 0Y6T0Z2, 0Y6T0Z3, 0Y6U0Z0, 0Y6U0Z1, 0Y6U0Z2, 0Y6U0Z3, 0Y6V0Z0, 0Y6V0Z1, 0Y6V0Z2, 0Y6V0Z3, 0Y6W0Z0, 0Y6W0Z1, 0Y6W0Z2, 0Y6W0Z3, 0Y6X0Z0, 0Y6X0Z1, 0Y6X0Z2, 0Y6X0Z3, 0Y6Y0Z0, 0Y6Y0Z1, 0Y6Y0Z2, 0Y6Y0Z3 |
|  |  | Exclusion ICD 10 procedure for traumatic amputation |  | S98011A, S78012A, S98012A, S78019A, S98019A, S78021A, S98021A, S78022A, S98022A, S78029A, S98029A, S78111A, S98111A, S78112A, S98112A, S78119A, S98119A, S78121A, S98121A, S78122A, S98122A, S78129A, S98129A, S78911A, S98131A, S78912A, S98132A, S78919A, S98139A, S78921A, S98141A, S78922A, S98142A, S78929A, S98149A, S88011A, S98211A, S88012A, S98212A, S88019A, S98219A, S88021A, S98221A, S88022A, S98222A, S88029A, S98229A, S88111A, S98311A, S88112A, S98312A, S88119A, S98319A, S88121A, S98321A, S88122A, S98322A, S88129A, S98329A, S88911A, S98911A, S88912A, S98912A, S88919A, S98919A, S88921A, S98921A, S88922A, S98922A, S88929A, S98929A |
